# Editorial: Animals and social interaction

**DOI:** 10.3389/fsoc.2026.1927239

**Published:** 2026-07-23

**Authors:** Mika Simonen

**Affiliations:** Faculty of Social Sciences, University of Helsinki, Helsinki, Finland

**Keywords:** animal actions, animal cognition, emotional burden, empathy, expectation, human–animal interaction, intersubjectivity, motivation

This Research Topic is a showcase of contemporary approaches to the study of “*Animals and social interaction*.” The seven contributions included here span a broad continuum of perspectives. They begin with human informants and their understandings of animals, move to the middle ground of human–animal interaction, where there are no informants but rather a focus on the interactional space and boundaries between species (Peltola and Simonen, 2024), and finally arrive at animal participants who offer insights into social interaction both indirectly and directly.

## 1 Toward animal social interaction

The broadest umbrella concept for the study of human and non-human animals is human–animal studies (HAS), which encompasses not only social interaction but also cultural, ethical, political, philosophical, and environmental perspectives (Deleuze and Guattari, 1987/2005; DeMello, 2021; Derrida, 2005; Haraway, 2003, 2008, 2016). Within this broader framework, human–animal interaction (HAI) focuses specifically on interactions between humans and animals and can be regarded as a nested concept covering all forms of such interaction (e.g., Shoesmith et al., 2025). Finally, *Animal Social Interaction* (ASI) represents a more specific approach that emphasizes the social-relational dimension of human–animal interaction.

Investigating the relationships between human and non-human animals in social interaction was the central aim of this Research Topic. The field of ASI—a term used here to distinguish it from human social interaction, rather than to deny that humans are animals—has advanced considerably in recent years. In particular, researchers have increasingly examined how non-human animals actively participate in social interactions and how humans contribute to these interactional processes. Although many studies adopt a predominantly human-centered perspective, they nevertheless contribute substantially to our understanding of ASI (Bergmann, 1988; Mitchell, 2001; Tannen, 2004; Roberts, 2004; Goode, 2006; Laurier et al., 2006; Goodwin, 2009; Jerolmack, 2009; Logue and Stivers, 2012; Rossano, 2013; MacMartin et al., 2014; Rossano and Liebal, 2014; Simonen, 2017; Mondada, 2018; Mondémé, 2018; Peltola, 2018; Pika et al., 2018; Cornips, 2019, Mondémé, 2020; Cornips and van den Hengel, 2021; De Malsche and Cornips, 2021; Due, 2021; Harjunpää, 2021; Lohi and Simonen, 2021; Simonen, 2021; Simonen and Lohi, 2021; Cornips, 2022; Harjunpää, 2022; Mondada and Meguerditchian, 2022; Mondémé, 2022; Arathoon, 2023; Cornips et al., 2023; Due, 2023; Mitchell, 2023; Mondémé, 2023a,b,c; Peltola, 2023; Rossano, 2023; Simonen, 2023; Szczepek Reed, 2023a,b; Cornips and van Koppen, 2024; De Rijk and Cornips, 2024; Jerolmack et al., 2024a,b; Laurier and Arathoon, 2024; Mondémé, 2024; Nilsson and Norrthon, 2024; Peltola and Simonen, 2024; Cornips, 2025a,b; Cornips and Wels, 2025; Harjunpää and Szczepek Reed, 2025a,b; Ikonen and Pehkonen, 2025; Jaworski and Jerolmack, 2025; Jääskeläinen, 2025; Mondémé, 2025; Norrthon and Nilsson, 2025; Ogden and Keevallik, 2025; Peltola and Grandgeorge, 2025; Szczepek Reed, 2025; Szczepek Reed and Lundesjö Kvart, 2025; Jääskeläinen, 2026).

**Figure 1:**
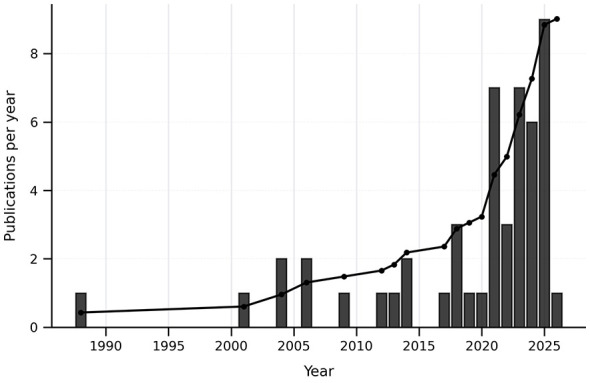
Distribution of publications contributing to the field of ASI between 1988 and 2026 (*n* = 65).

Although Figure 1 is not based on a systematic review of the literature and undoubtedly omits relevant publications, it nevertheless illustrates how the cited works are distributed across the period from 1988 to 2026. Overall, the figure demonstrates the rapid growth of publications in the field during recent years.

## 2 Contributions to the field

The contributions to this Research Topic provide diverse perspectives on “*Animals and social interaction*.” Two studies focus on human understandings of animals and engagement with animal-related issues. For example, one examines perceptions of animal ownership and the ways animals may challenge owners' expectations (Anderson et al.), while another investigates motivations for engaging with animal welfare organizations involved in animal rescue (Lu et al.). Using methods such as surveys, thematic analysis, and structural equation modeling, these studies illuminate human perspectives on animals and human–animal relationships.

Other contributions highlight how animals' physical conditions can shape interaction and human wellbeing. Pryjmak, Zablotski, Lauer found that owners of dogs with chronic paresis or paralysis reported greater emotional burden than owners of dogs with limb amputations (Pryjmak, Zablotski, Mille et al.). Based on online survey data, these studies demonstrate how animals can influence human emotional experiences, generating both positive feelings and concerns related to health and mobility.

Beyond human perspectives, one contribution examines the intersubjective organization of human–animal interaction (Peltola and Simonen, 2024; Simonen and Lohi, 2021). Applying intersubjectivity as an analytical concept to historical research, Taşdizen et al. trace human–animal coexistence from contemporary practices back to the sixteenth century. In this study, intersubjectivity is understood through the temporal dimensions of openness and relationality, extending earlier interactional approaches that examined intersubjectivity through turn-taking (Heritage, 1984).

Two studies place greater emphasis on animals' participation. An experimental study measured animals' cortisol levels before and after encounters with humans and found lower concentrations in later meetings, suggesting that repeated interaction became increasingly familiar and less stressful (Artemiou et al.). While providing only indirect access to animals' experiences, the findings demonstrate that human participation influences animal wellbeing in interaction.

A second study analyzes video recordings collected using GoPro cameras attached to dogs (Simonen). Focusing on ball-throwing activities during dog walks, the analysis shows how dogs initiate interaction and how cognition emerges through the sequential organization of actions. The findings are discussed in relation to animal cognition research (Merritt, 2021).

Together, the seven contributions advance our understanding of ASI through four complementary approaches: surveys, historical investigations, experimental studies, and analyses of naturally occurring interactions. Collectively, they demonstrate the value of examining how different species orient to one another in interspecies social life.

## 3 Final considerations

A central message for future studies of ASI is the need to recognize the limits of human knowledge about non-human animals. Future research should focus on concrete interactional encounters between humans and non-human animals. One way of conceptualizing such encounters is through the notion of a trading zone (Collins et al., 2007), understood as an interspecies space in which participants coordinate actions and negotiate shared understandings despite species-specific differences. Participation in these encounters requires adaptation from both humans and non-human animals. As Collins et al. (2007) argue, interaction often depends on forms of interactional expertise that make communication across different communities possible. In interspecies interaction, this process is not limited to humans. Social interaction between humans and non-human animals is therefore neither exclusively human-led nor exclusively animal-led; rather, it emerges through the joint accomplishment of participants who continuously orient to one another and to the interactional demands of the situation (Simonen and Lohi, 2021, p. 424–425).

Ultimately, the study of ASI should move beyond explaining animals solely through human categories and assumptions. Instead, it should seek to understand how different species mutually organize their relationships and activities in everyday life. By focusing on interaction itself, researchers can better understand how humans and non-human animals collaboratively create shared social worlds.
